# Navigating Treatment Selection in Acute Severe Ulcerative Colitis: A Prospective Mixed Method Analysis of Factors Influencing Patient Decision Making

**DOI:** 10.21203/rs.3.rs-9308389/v1

**Published:** 2026-04-15

**Authors:** Reid Herran, Samuel Wilcox, Gal Hodish, Syed A. Hassan, Haley Mertens, Christopher V. Almario, Philip Gu, Maia Kayal, Shirley Cohen-Mekelburg, Randolph E. Regal, Shrinivas Bishu, Corey A. Siegel, Melissa DeJonckheere, Peter D.R. Higgins, Jeffrey A. Berinstein

**Affiliations:** University of Michigan; University of Michigan; University of Michigan; University of Michigan; University of Michigan; Cedars-Sinai Center for Outcomes Research and Education (CS-CORE); Cedars-Sinai Medical Center; Icahn School of Medicine at Mount Sinai; University of Michigan; University of Michigan; University of Michigan; Dartmouth Hitchcock Medical Center; University of Michigan; University of Michigan; University of Michigan

**Keywords:** acute severe ulcerative colitis, ulcerative colitis, Janus Kinas Inhibitors, Upadacitinib

## Abstract

Purpose: Acute severe ulcerative colitis (ASUC) is a life-threatening medical emergency necessitating immediate hospitalization and rapid initiation of therapy. For decades, intravenous corticosteroids (IVCS) have remained first-line, with rescue therapies reserved for corticosteroid-refractory patients. However, the therapeutic landscape has evolved, shifting towards earlier initiation of advanced therapy. This study analyzes factors influencing patient decision making for treatment of ASUC. Methods: In this prospective, mixed-methods study, infliximab- and JAK-inhibitor-naïve patients with ASUC were enrolled. Participants were presented with educational resources on IVCS, upadacitinib, and infliximab, then asked to choose between continuing IVCS or early initiation of advanced therapy. Factors influencing their preferences were captured via semi-structured interviews, and clinical outcomes were followed. Results: Eleven patients were prospectively enrolled. Analysis of semi-structured interviews revealed five dominant themes influencing treatment decision-making: (1) reliance on physician expertise, (2) contextualized treatment selection, (3) concrete risk assessment, (4) empathetic individualization, and (5) a strong preference for oral therapy. Notably, we observed significant discordance between patient treatment preferences and medication initiated following discussion between the participant and their primary clinical team, particularly among participants favoring upadacitinib. Conclusion: These five principal themes offer actionable opportunities to assist patients in making well-informed decisions regarding ASUC therapy. Clinicians should utilize these insights to develop evidence-based patient decision aids that specifically address patient priorities. While patients often prioritize oral therapy, their choices often diverge from the therapy initiated after discussing with their primary clinical team. Future efforts should focus on bridging this discordance by integrating patient priorities with available evidence into the clinical decision-making process.

## Introduction

Ulcerative colitis (UC) is a chronic immune-mediated inflammatory condition of the colon that presents with fecal urgency, diarrhea, rectal bleeding, and abdominal pain [1]. In recent years, multiple effective treatments have been approved for UC, significantly lowering symptom severity and complications such as the need for surgery, hospitalization, and death [2,3]. Despite medical advancements, 25–50% of UC patients will develop acute severe ulcerative colitis (ASUC), a severe flare often requiring hospitalization, during the course of their disease [4].

ASUC is a life-threatening presentation of UC that necessitates prompt hospitalization for rapid diagnostic work-up and treatment [5,6]. IV corticosteroids have been first-line for ASUC for over 70 years, which fail to prevent colectomy in 30% of patients [7]. While rescue therapies like infliximab and cyclosporine provide further salvage options, 30% of steroid-refractory patients still ultimately require a colectomy [8–11]. Recently, Janus kinase inhibitors (JAKi), such as tofacitinib and upadacitinib, have emerged as additional promising therapeutic options for patients with steroid-refractory ASUC [12–15].

Each of these medical treatments possesses unique benefits and risks in terms of speed of onset, shortterm and long-term efficacy, potential side effect profile, route of administration, ease of use, and required pre-treatment and on-treatment monitoring. In the absence of direct head-to-head clinical trials comparing these therapies, and with a lack of robust predictors for treatment response, patients and clinicians have limited evidence-based guidance on which agent to select. This uncertainty represents a major shortfall in our approach to ASUC, as timely effective treatment initiation is critical to prevent severe complications such as toxic megacolon, perforation, urgent colectomy, postoperative complication, and mortality [16,17).

Given these clinical limitations, understanding the patient factors that drive therapy selection during this critical window is essential for navigating treatment complexities and delivering tailored, patientcentered care. Furthermore, engaging patients in shared decision-making fosters a sense of empowerment, which is strongly associated with improved treatment adherence, satisfaction, and outcomes [18,19]. Insights from this process can help identify communication gaps, informing the development of targeted patient-facing materials and decision aids. Therefore, this study aims to examine the key factors that influence patient decision-making when selecting therapies for ASUC.

## Methods

### Study Design

We employed a prospective, convergent mixed methods design to gain a better understanding of treatment decision-making in hospitalized patients with ASUC. To achieve this, we integrated qualitative data from in-depth semi-structured interviews with quantitative measures, including standardized engagement surveys and 30-day clinical outcomes. To ensure the findings reflect real-time clinical decisions rather than theoretical preferences, ASUC patients were enrolled within 24–48 hours of admission and prior to advanced or rescue therapy initiation. This approach aimed to capture the lived perspectives and cognitive processes of patients during the window where high-stakes therapy selection occurs in a real-world setting. In this study we utilized a hybrid approach combining deductive coding (applying pre-determined themes based on clinical experience) with inductive coding (allowing new themes to emerge directly from patient interviews) [20].

### Participants and Recruitment

Adult patients (≥ 18 years-old) hospitalized with an ulcerative colitis flare at the University of Michigan from February 2025 to January 2026 were screened for eligibility. Patients were eligible for inclusion if they had an established diagnosis of UC, met Truelove and Witts’ criteria for ASUC, and were anti-TNF and JAK-inhibitor naïve. Patients were excluded if inpatient advanced or rescue therapy (e.g., infliximab, upadacitinib, tofacitinib, or cyclosporine) had already been initiated. Truelove and Witts’ criteria for ASUC was defined as ≥6 bloody bowel movements per day plus at least one of the following: 1) temperature >37.8°C, 2) heart rate >90 bpm, 3) hemoglobin <10.5 g/dL, 4) erythrocyte sedimentation rate (ESR) >30 mm/hr, or 5) C-reactive protein (CRP) > 3.0mg/dL [21].

We identified eligible patients through automated electronic health record (EHR) alerts for patients seen in the emergency room or hospital with a diagnosis of the UC and through daily review of the inpatient gastroenterology team census. Eligible participants were approached in person and enrolled following IRB-approved written informed consent.

### Data Collection and Procedures

A study team member not involved in the patient’s direct clinical care presented educational material, conducted semi-structured interviews, and completed surveys with enrolled participants. Prior to the qualitative assessment, a member of the study team delivered a standardized, interactive PowerPoint presentation. This session covered the natural history of ASUC, potential complications, and the clinical rationale for urgent therapy initiation. Detailed comparisons of three potential primary treatment options (intravenous corticosteroids, infliximab, and upadacitinib) were presented, with a specific focus on their respective mechanisms, efficacy of treatment, route of administration, strength of data in the ASUC setting, safety profiles, and outpatient medication access. The presentation was designed to be interactive with regular pauses to elicit participant feedback on the material, clarify concepts, and encourage real-time questions and interruptions. The educational presentation was developed leveraging user-centered design principles and evidence-based health literacy strategies [22–24]. This was pilot tested with patients prior to deployment and was iteratively refined throughout the study [25]. Feedback obtained during pre-deployment testing and early interviews was used to modify subsequent versions of the presentation, allowing for real-time improvements in clarity, tone, and delivery (see https://docs.google.com/presentation/d/1l5O7v5RlIWy2IvlCgMnU-iOFRKQBICTbjF_Z7Psi30w/edit?usp=sharing for final version).

Following the educational presentation, the study team member conducted a 30- to 45-minute semistructured interview. The interview was audio-recorded and followed an interview guide (Supplemental Figure 1) designed to explore patient perspectives on ASUC treatment selection and key factors contributing to their individual therapy decisions. The flexible nature of the interview allowed for an indepth exploration of unique patient backgrounds and personal values while maintaining focus on these core domains.

To complement the qualitative interviews, we utilized the Patient Preferences for Engagement Tool 13item Short Form (PPET-13), a standardized, validated, objective measure of the degree to which patients desired to be involved in their clinical decision-making [22,26]. This validated tool allowed us to quantify patient-centered priorities and engagement preferences in a standardized manner. Following the educational intervention and interview, the patient’s elicited preferences were conveyed to the primary clinical gastroenterology team, who maintained ultimate responsibility for final therapeutic selection and clinical management.

Participants were followed for 30 days post-discharge to track their clinical course and outcomes as well as changes in PPET-13 scores overtime. We performed a comprehensive chart review to determine the ultimate therapeutic selection and if the actual therapeutic agent initiated aligned with the patient’s expressed preference during our interview, treatment persistence, and colectomy status. Demographic information (e.g., age, sex, race, ethnicity), social factors (e.g., highest level of education, marital status), and disease-characteristics (e.g., disease duration, extent) were extracted from the EHR when available and confirmed directly with the patient during the enrollment window if needed.

### Statistical Analysis

Patient interviews were analyzed using an established hybrid inductive-deductive approach [29]. This methodology ensured that findings were grounded in both existing clinical frameworks (deductive coding) while capturing emerging unanticipated themes directly from participant interviews (inductive coding). To ensure consistency and validity of our qualitative findings, each interview was independently coded by at least two study team members (RH, SW, GH, JB). The research team met regularly to resolve coding discrepancies through consensus and to refine the final thematic map. Representative quotes were extracted to illustrate the core perspectives within each theme. Quantitative data were analyzed using descriptive statistics. Medians and interquartile ranges (IQR) were presented for continuous variables and frequencies with percentages were presented for categorical variables. Quantitative data were compared across treatment selection groups to identify potential trends or patterns in decisionmaking. All quantitative analyses were performed using R Statistical Software (version 4.4.1; R Foundation for Statistical Computing, Vienna, Austria). For mixed methods analysis, qualitative themes were compared to quantitative results by domain.

## Results

### Patient Demographics and Clinical Characteristics

Of the 14 eligible patients hospitalized with ASUC approached about study participation, 11 patients enrolled. The median age of our cohort was 25 years (IQR: 22, 68). The population was predominantly White (82%) and male (64%), and all participants identified as non-Hispanic. At the time of admission, 73% of the total cohort was already utilizing corticosteroids. At the time of admission, prior exposure to advanced therapies was low, however the cohort exhibited high objective markers of disease severity at with a median albumin of 3.50 g/dL (IQR: 3.00, 3.70), median CRP of 5.80 mg/dL (IQR: 2.00, 8.70), median fecal calprotectin of 2,072 mg/kg (IQR: 860, 3,500), with 73% of patients presenting with a Mayo Endoscopic Score of 3 (Table 1)

### Qualitative Findings: Patient Perspectives on ASUC Treatment Selection

Thematic analysis of the semi-structured interviews revealed five dominant themes regarding the decision-making process during hospitalization for ASUC: (1) Reliance on Physician Expertise (2) Contextualized Treatment Selection, (3) Concrete Risk Assessment, (4) Empathetic Individualization, and (5) Strong Preference for Oral Therapy (Table 2).

### Theme 1:Reliance on physician Experties

Thematic analysis revealed that when presented with various treatment pathways, nearly all participants prioritized the GI physician’s expert opinion, although the degree of importance varied slightly by patients. One patient described the provider as the “main supporter of helping [them] make the right decision,” while another explicitly stated a preference for the physician to take on the “majority role” in treatment selection decision-making, provided there was a perceived thoroughness in the medical examination and review of the patient’s history.

Patients described it as “disconcerting” to be presented with a list of options without accompanying professional guidance, largely due to their own lack of formal expertise in this area. This sentiment was exemplified by a patient who noted “it is always a little disconcerting when they lay all the options on the table and go… ‘which one do you want?’ I didn’t go to med school!” In contrast, one patient felt most empowered when allowed to make treatment decisions with the support of both her provider and family, emphasizing the value of having her final wishes respected within a shared-decision making framework where she ultimately had the final say in selecting a therapy.

These qualitative perspectives are directly supported by the PPET-13 quantitative results. While 100% of participants “Agreed” or “Strongly Agreed” that they wanted to be involved in their healthcare, their preference for independent decision-making was notably neutral, with a median score of 3.0 and only 36% (4/11) agreeing that they preferred to make decisions on their own. In stark contrast, the desire to make decisions with a healthcare provider was universally high, with a median score of 5.0. All 11 participants (100%) “Agreed” or “Strongly Agreed” that they preferred a collaborative approach with their provider. Notably, 73% (8/11) “Strongly Agreed” with this collaborative model, while not a single patient (0%) “Strongly Agreed” that they should make decisions entirely by themselves.

### Theme 2: Contextualized Treatment Selection

Participants had a strong preference for treatment discussions to be centered around their unique clinical circumstances and lifestyle priorities. For example, a patient with a comorbid neurologic disorder emphasized the need for a multidisciplinary, “holistic approach” that takes into account both conditions. He noted that having a care team able to assess the “pros and cons” relative to his specific case was ideal, stating, “you’ve gotta get everybody involved… that’s what I appreciated.”

Lifestyle constraints and social roles served as primary drivers for treatment selection. A high-level athlete highlighted that a demanding travel schedule made an oral option more appealing than coordinating frequent recurring infusions. Similarly, a young mother prioritized therapies that would facilitate a rapid return to her caregiving role, citing understanding how the medication would “align with [her] life already, and what changes [she was] going to have to make” as the most important factor when selecting a medication. Another participant, while valuing traditional medical therapies, sought to couple it with diet, gut-directed behavioral therapies, and “natural” therapies, emphasizing a desire for a multimodal approach that allowed the body to heal rather than “just treating symptoms.”

These qualitative insights are reinforced by the PPET-13 results, which indicated that participants placed a high value on collaborative planning, goal setting, ensuring the care plan is followed, and incorporating feedback into their care and treatment selection. 91% (10/11) of participants “Strongly Agreed” that they should be involved in setting their own health goals suggesting that patients have very specific outcomes in mind. Most patients “Agreed” or “Strongly Agreed” that they should be involved in creating their own care plan, with 64% (7/11) providing the highest possible score, suggesting that patients have a strong desire to help build their contextualized “map” to get to those goals. 100% of participants “Agreed” or “Strongly Agreed” that their healthcare-related feedback should be heard, suggesting that participants want their lived experience (e.g., the burden of infusions or inability to care for their loved ones) to be taken seriously by the medical team and factored into their proposed treatment plans.

### Theme 3: Concrete Risk Assessment

Participants expressed a strong desire for transparent, categorized, and quantitative data regarding the potential adverse effects of proposed therapies. Thematic analysis revealed that patients often felt overwhelmed by the “laundry list” of “a gazillion side effects” typically presented in medical advertisements and sought a more structured understanding of risk. One patient highlighted the necessity of understanding the “odds”, questioning whether a specific risk applied to “0.01% of the population or 50% of the population” noting that patients are effectively being asked to “risk their health without really knowing exactly what the odds are.”

Several participants expressed a preference that the presentation of adverse event risk data prioritize clarity over exhaustive detail. One participant suggested organizing information into “categories of types of problems” to show patterns as opposed to listing “every single one [potential side effect].” Furthermore, some participants argued that providing specific percentages was not only helpful but necessary to provide a realistic sense of scale. One patient noted that seeing “pretty high” percentages might be beneficial to “scare [patients] a little” serving as a vital alert to more probable risks.

In contrast, some participants felt that specific percentages were less critical than the clinical safety net provided by the physician and the required pre-treatment and intra-treatment screening and monitoring for infections such as tuberculosis and hepatitis. Others noted that while they valued knowing the percentages, such data would not ultimately dictate their decision, as adverse reactions remain inherently “unpredictable” on the individual level.

These qualitative insights are supported by the high priority participants placed on being fully informed about their care options within the PPET-13 survey. 91% (10/11) of participants “Agreed” or “Strongly Agreed” that they should be informed about the advantages and disadvantages of each option, while 100% of participants “Agreed” or “Strongly Agreed” that it is important to know everything about their health.

### Theme 4: Empathetic Individualization

Participants emphasized that effective decision-making is predicated on a personalized relationship with the care team, which should be rooted in empathy and transparency. There was a clear misalignment between a patient’s lived experience and the provider’s understanding of the physical and emotional impact UC had on the patients they were caring for. Participants frequently noted that a provider’s reliance on general clinical experience often came at the expense of individual empathy. One patient articulated this disconnect sharply, stating: “You have no idea what I’m going through here… You are just going by your experience of dealing with other patients… and they won’t pertain to me.” However, this same patient reported high satisfaction with the care he received during his current hospitalization. He attributed this positive experience to having a care team consisting of multiple doctors at various career stages which served as an important vehicle to ensure no details were missed, and the patient’s concerns were repeatedly being heard.

A critical component of this individualization was the rejection of paternalistic “dictating.” One participant described a negative experience where a team insisted on a single “best option” without considering the patient’s lived reality, noting, “I get that, but it’s just not going to work for me.” Instead, patients advocated for a “joint approach” characterized by complete transparency regarding the benefits, negatives, and clinical justifications for each treatment.

For an elderly participant, the hallmark of quality care was a physician’s willingness to hold an unhurried discussion that accounted for his specific comorbidities. As one participant summarized, the goal is for providers to “keep it real,” “be straightforward” and “transparent,” and provide honest guidance so they do not feel “manipulated” into a decision. One participant described a negative experience during a prior hospitalization in which his inpatient team “didn’t really seem to understand. They were like ‘no’ [when referring to a patient-proposed treatment], this is what you got to do.”

These qualitative perspectives on the necessity of an empathetic, personalized connection are reinforced by the high priority participants placed on collaborative oversight in the PPET-13 survey. 100% of participants “Agreed” or “Strongly Agreed” that their healthcare provider should check with them to see how they feel about a decision, while 100% of participants also “Agreed” or “Strongly Agreed” that they should be given the opportunity to ask all their questions about their health.

### Theme 5: Strong Preference for Oral Therapy

Thematic analysis revealed a significant preference for oral small molecules over traditional biologic infusions, primarily driven by the perceived convenience and long-term sustainability of the treatment. Following the educational presentation, 64% (7/11) of patients selected upadacitinib. The convenience of oral administration was the dominant factor in this selection; one patient noted that “taking a pill would be very advantageous” as it avoids the logistical burdens of injections, infusions, or need for temperature-regulated medication storage. Participants emphasized that for a chronic condition requiring long-term therapy, the “ease of getting it and ease of taking it” were critical determinants of their preference.

In contrast, 27% (3/11) of patients who preferred the infliximab pathway cited different priorities. These participants valued the “longer track record” and established familiarity with the anti-TNF medication class. Furthermore, continued outpatient access following discharge and safety profiles, specifically regarding safety in pregnancy, were noted as deciding factors for those opting for the biologic infusion over the novel oral therapy. Several patients noted that while they preferred the oral option, the decision “ultimately comes down to” insurance coverage and out-of-pocket costs, suggesting that financial information is a necessary component of informed medication selection.

### Evaluation of the Patient Decision Aid

Participants provided specific feedback on the PowerPoint-based decision aid, which was used to facilitate the iterative refinement of the tool. Participants overwhelmingly preferred a more comprehensive approach to information, expressing a desire to be fully informed about their expected disease course and potential complications. While some participants found text slides helpful for detail, the majority of patients felt that “words were hard to interpret” during an acute flare and strongly advocated for easier to understand diagrams and illustrations.

### Treatment Selection and Administration Discordance

Following the educational intervention, one patient (9%) elected to remain on IV corticosteroids alone (10%), while 3 (27%) patients selected infliximab and 7 (63%) patients selected upadacitinib in addition to continuing IV corticosteroids. Overall, 45% (n=5) of the total cohort received their initial selection. Discordance between the initial selection and final administration was more frequent in the upadacitinib group (71% discordance) compared to the infliximab group (23% discordance).

This shift in therapy selection primarily occurred following a subsequent shared discussion between the patient and the primary gastroenterology team. In 67% of these cases, the final treatment plan was adjusted based on clinical consultation with the attending physician. These adjustments were often driven by the provider’s assessment of current standards of care and guidelines, the strength of available evidence for specific agents, or personal experience. Additional factors contributing to the final treatment selection discordance included patient-driven concerns for insurance-related access issues (17%) and shifts in patient preference (17%).

### Patient Engagement Preferences

Overall, baseline PPET-13 scores demonstrated a high desire for engagement in clinical decision-making, with a median of score 56.0 (IQR: 53.0, 61.0), which remained stable at the 30-day follow-up. The group that selected upadacitinib showed the highest increase in engagement preference over time (Median change: +2.0), whereas those selecting infliximab or corticosteroids alone showed slight decreases (−2.0 and −1.0, respectively).

## Discussion

This mixed-methods study provides valuable insight into the mindset of patients facing treatmentselection decisions for ASUC. When making real-time decisions, patients demonstrated a nuanced “paradox of autonomy.” Our findings suggest that patients overwhelmingly desire to be the primary stakeholders in their care, however, remain reliant on physician expertise (Theme 1) when navigating the complexity of ASUC. This duality is well-documented in high-acuity settings across other medical fields, where patients often seek more information but paradoxically prefer to delegate the final high-stakes choice to a trusted clinician as illness severity increases [23]. In this high-acuity setting, expert guidance from an experienced physician remains the most important resource to patients, yet this reliance is not unconditional. Patients sought out empathetic individualization (Theme 4) suggesting that patients explicitly rejected “clinical detachment” and “paternalistic” unilateral decision making, instead seeking an empathy-driven dialogue where their unique clinical and social contexts are recognized (Theme 2).

We observed a notable therapeutic discordance between the medications patients initially selected and the treatments they ultimately received, with only 45% of the total cohort receiving their original selection. While physician disagreement accounted for 67% of the discordant cases, the discrepancy was not related to a dismissal of patient preferences. Rather, the final treatment plan was adjusted based on joint decision making between the primary treatment team and the patient, reflecting the complex clinical decision making between patient-centered preferences and the primary gastroenterology team’s expert guidance. This observation is consistent with Theme 1, where patients emphasized the importance of making high-stakes decisions with input from an expert clinician.

Therefore, the therapeutic discordance should be viewed as the fulfillment of the patient’s request for professional expertise as the educational intervention was designed to be a balanced, neutral resource, providing the data necessary for informed preference but not providing prescriptive guidance.

While 64% of patients favored the convenience of oral therapy in their long-term treatment pathway (Theme 5), patients were frequently started on infliximab in the hospital for ASUC following discussion and reconciliation with the clinical treatment team. This preference for oral therapy mirrors findings in broader IBD out-patient populations, where up to 87% of patients express a preference for oral administration due to perceived freedom and ease of use [24,25]. This preference for infliximab likely reflects the provider’s assessment of current standards of care and guidelines, the strength of available evidence for specific agents, concerns about particular safety risks, or personal experience. This discrepancy between patient and physician preference and expectations is common but may have a detrimental impact on treatment selection and adherence [27,28].

Theme 3 (“Concrete Risk Assessment”) highlights that ASUC patients sought specific percentages and categorized adverse event profiles to provide a realistic sense of objective risk. This aligns with our feedback on the decision aid, where patients advocated for more diagrams and less text during the acute stress of a flare. Intriguingly, despite a clearly outlined absence of robust clinical trials supporting upadacitinib specifically for ASUC, a weakness explicitly disclosed in our presentation, patients felt there was sufficient evidence to support its use based on its established efficacy in moderate-to-severe UC. This willingness to embrace novel therapies aligns with prior research highlighting that anticipatory guidance and the utilization of decision aids, coupled with a reliance on physician expertise (Theme 1), are pivotal in navigating medical uncertainty [29,30]. This willingness to embrace novel therapies aligns with the Health Belief Model, which posits that a patient’s motivation to accept a treatment is driven by the “perceived severity” of their illness and the “perceived benefits” of the intervention [31,32]. In the highacuity setting of ASUC, our findings suggest that when patients experience a significant symptom burden and are faced with the imminent threat of surgery, they may prioritize the theoretical advantage of a rapid-acting novel mechanism over the comfort of established therapies with slower onset or known limitations [29,33].

While patients may prioritize potential efficacy and convenience, physicians often maintain a more nuanced, and perhaps more conservative, interpretation of how medical literature generalizes to an individual patient. This is notably illustrated by the fact that only one patient selected IV corticosteroids alone—the treatment pathway with the most historical data—emphasizing that patients overwhelmingly prioritize colon preservation and are willing to initiate advanced therapies early rather than awaiting a 2472 hour steroid-response assessment [29].

Our findings support a joint approach to ASUC management, where tailored decision aids provide the anticipatory guidance patients require to feel empowered. Looking ahead, our findings suggest that clinicians can enhance patient care by embracing shared decision-making that honors each individual’s unique clinical path and health history. By moving toward a more collaborative partnership, clinicians can empower patients to navigate complex choices with confidence and personalized support. Table 3 provides proposed actions based on major themes identified.

Several limitations warrant consideration: First, the small cohort (n=11) limits the generalizability of the quantitative findings. However, for the qualitative component of this research, we observed that thematic saturation was reached within this cohort. The final interviews yielded minimal novel themes regarding patient preferences or barriers to shared decision-making, suggesting the sample size was sufficient to capture the breadth of perspectives within this specific ASUC population. Second, our study design may be affected by selection and responder bias. Our results do not capture the perspectives of the three patients who declined participation. The majority of these individuals cited a reluctance to initiate any UC-related advanced therapy as their major reason for not participating, suggesting a potential responder bias toward those already open to advanced therapy, although this view was rare, with only 3 of the 14 (21%) patients approached for the study declining participation. In addition, there is potential for investigator bias, wherein the researcher’s own clinical perspectives or cognitive biases might have inadvertently influenced the patient education materials or the thematic elicitation during interviews. To mitigate this, the educational materials were developed with a specific emphasis on neutrality and balance, and the content underwent iterative modification based on direct patient feedback and rigorous pre-implementation testing specifically designed to identify and reduce bias. Finally, the findings of this study may reflect the specific clinical environment of our institution, where there is a high institutional familiarity with upadacitinib. While every effort was made to present all therapeutic options using neutral, unbiased educational materials, the local standard of care and clinician comfort using upadacitinib likely contributed to our findings.

In conclusion, patients hospitalized for ASUC demonstrate a high desire for engagement in their care, which was characterized not by a request for total autonomy, but by a preference for an empathy-driven, collaborative partnership with their clinical team. While patients highly prefer oral therapies for their convenience (64%) and seek quantitative, categorized risk assessments, a significant discordance existed between participant medication selection and the actual medication received. This gap, where 67% of patient selections were overridden by physician disagreement, highlights the possible discomfort physicians have with newer treatments such as JAK-inhibitors which have less robust clinical evidence at this time. Despite the absence of robust prospective data for newer therapies in the ASUC setting, patients are willing to trial medications with theoretical advantages to avoid surgery, prioritizing colon preservation over traditional steroid-response wait times.

## Supplementary Material

Supplementary Files

This is a list of supplementary files associated with this preprint. Click to download.

- InterviewGuideSupplementalFigure1.pdf

## Figures and Tables

**Table 1:** Patient Demographics, Clinical Characteristics, Clinical Course and Outcomes

	Overall	IV corticosteroids (N = 1)	IV corticosteroids + Infliximab (N = 3)	IV corticosteroids + Upadacitinib (N = 7)
**Age (years)**	25 (22, 68)	33	25 (22, 68)	25 (22, 70)
**Female**	4 / 11 (36%)	1 / 1 (100%)	2 / 3 (67%)	1 / 7 (14%)
**Race**				
White	9 / 11 (82%)	1 / 1 (100%)	3 / 3 (100%)	5 / 7 (71%)
Black	2 / 11 (18%)	0 / 1 (0%)	0 / 3 (0%)	2 / 7 (29%)
**Non-Hispanic**	11 / 11 (100%)	1 / 1 (100%)	3 / 3 (100%)	7 / 7 (100%)
**Education Level**				
Less than high school	0 / 11 (0%)	0 / 1 (0%)	0 / 3 (0%)	0 / 7 (0%)
High school graduate	5 / 11 (45%)	0 / 1 (0%)	1 / 3 (33%)	4 / 7 (57%)
College graduate	2 / 11 (18%)	0 / 1 (0%)	1 / 3 (33%)	1 / 7 (14%)
Master’s or professional graduate	3 / 11 (27%)	1 / 1 (100%)	1 / 3 (33%)	1 / 7 (14%)
Trade school graduate	1 / 11 (9.1%)	0 / 1 (0%)	0 / 3 (0%)	1 / 7 (14%)
**Employment Status**				
Employed	6 / 11 (55%)	1 / 1 (100%)	2 / 3 (67%)	3 / 7 (43%)
Unemployed	1 / 11 (9.1%)	0 / 1 (0%)	0 / 3 (0%)	1 / 7 (14%)
Retired	2 / 11 (18%)	0 / 1 (0%)	0 / 3 (0%)	2 / 7 (29%)
Student	2 / 11 (18%)	0 / 1 (0%)	1 / 3 (33%)	1 / 7 (14%)
**Marital Status**				
Single	6 / 11 (55%)	0 / 1 (0%)	1 / 3 (33%)	5 / 7 (71%)
Married	4 / 11 (36%)	1 / 1 (100%)	2 / 3 (67%)	1 / 7 (14%)
Widowed	1 / 11 (9.1%)	0 / 1 (0%)	0 / 3 (0%)	1 / 7 (14%)
**Corticosteroid use on admission**	8 / 11 (73%)	0 / 1 (0%)	3 / 3 (100%)	5 / 7 (71%)
**Prior Infliximab Use**	0 / 11 (0%)	0 / 1 (0%)	0 / 3 (0%)	0 / 7 (0%)
**Prior Adalimumab Use**	0 / 11 (0%)	0 / 1 (0%)	0 / 3 (0%)	0 / 7 (0%)
**Prior Vedolizumab Use**	1 / 11 (9.1%)	0 / 1 (0%)	0 / 3 (0%)	1 / 7 (14%)
**Prior Ustekinumab Use**	1 / 11 (9.1%)	0 / 1 (0%)	0 / 3 (0%)	1 / 7 (14%)
**Prior Risankizumab Use**	0 / 11 (0%)	0 / 1 (0%)	0 / 3 (0%)	0 / 7 (0%)
**Prior Guselkumab Use**	1 / 11 (9.1%)	0 / 1 (0%)	1 / 3 (33%)	0 / 7 (0%)
**Prior Tofacitinib Use**	0 / 11 (0%)	0 / 1 (0%)	0 / 3 (0%)	0 / 7 (0%)
**Prior Upadacitinib Use**	0 / 11 (0%)	0 / 1 (0%)	0 / 3 (0%)	0 / 7 (0%)
**Admission Albumin (g/dL)**	3.50 (3.00, 3.70)	3.70 (3.70, 3.70)	2.50 (2.30, 3.40)	3.50 (3.20, 3.70)
**Admission CRP (mg/dL)**	5.80 (2.00, 8.70)	1.10 (1.10, 1.10)	6.20 (0.90, 9.00)	5.80 (3.30, 8.70)
**Admission Fecal Calprotectin (mg/kg)**	2,072 (860, 3,500)	3,500	860 (540, 3,500)	2,072 (1,125, 2,096)
**Endoscopic Mayo Score**				
Mayo 2	3 / 11 (27%)	0 / 1 (0%)	0 / 3 (0%)	3 / 7 (43%)
Mayo 3	8 / 11 (73%)	1 / 1 (100%)	3 / 3 (100%)	4 / 7 (57%)
**Baseline Engagement Score**	56.0 (53.0, 61.0)	56.0	56.0 (56.0, 63.0)	57.0 (53.0, 61.0)
**30-Day Follow-up Engagement Score**	59.0 (55.0, 62.0)	55.0 (55.0, 55.0)	57.0 (53.0, 61.0)	59.0 (55.0, 62.0)
**Change in Engagement Score**	1.0 (−2.0, 7.0)	−1.0 (−1.0, −1.0)	−2.0 (−3.0, 1.0)	2.0 (−2.0, 9.0)
**Received Selected Treatment**	5 / 11 (45%)	1 / 1 (100%)	2 / 3 (67%)	2 / 7 (29%)
**Reason Did Not Receive Treatment**				
Attending Did Not Agree	4 / 6 (67%)	-	1 / 1 (100%)	3 / 5 (60%)
Insurance-Related Access Issue	1 / 6 (17%)	-	0 / 1 (0%)	1 / 5 (20%)
Patients’ Preferences Changed	1 / 6 (17%)	-	0 / 1 (0%)	1 / 5 (20%)
**Colectomy**	4 / 11 (36%)	0 / 1 (0%)	1 / 3 (33%)	3 / 7 (43%)
**30-day Medication Persistence**	7 / 11 (64%)	1 / 1 (100%)	2 / 3 (67%)	4 / 7 (57%)

1 Median (Q1, Q3); n / N (%)

**Table 2:** Themes and Representative Quotes

		Representative Quote
**Theme 1**	Reliance on Physician Expertise	“It is always a little disconcerting when they lay all the options on the table and go, hey? Which one do you want? I didn’t go to med school. Make your argument, you know. Give me a good argument, and positive and negative. Right? Then, I can either buy your argument or not logically.”
**Theme 2**	Contextualized Treatment Selection	“like how the [neurologic disorder] and UC relate, and then the medication right? Having somebody look at the whole situation is ideal, and then them giving you their best assessment with the pros and cons.”
**Theme 3**	Concrete Risk Assessment	“So how likely are those, you know? Is this like 0.01 of the population? Or is this like 50% are going to have this, you know, because you’re asking people to make a choice and risk their health without really knowing exactly what the odds are.”
**Theme 4**	Empathetic Individualization	“I [would] just think to myself, you have no idea what I’m going through here. You don’t even ask me what’s going on. You are just going by your experience of dealing with other patients and passing that on to me, and they won’t pertain to me.”
**Theme 5**	Strong Preference for Oral Therapy	“Taking a pill would be very advantageous to just be able to take something and not to do any sort of injection or infusion, worry about keeping it cold… The ease of getting it and ease of taking it is important.”

**Table 3:** Proposed Actions Based on Major Themes Identified

		Proposed action
**Theme 1**	Reliance on Physician Expertise	After asking patient what is most important to them in their treatment plan, offer your professional opinion on the best path forward
**Theme 2**	Contextualized Treatment Selection	Medical complexities: consider consulting other subspecialties to help guide treatment decision Social complexities: take time to inquire about social support and commitments that may impact patient’s ability to realistically adhere to certain treatments
**Theme 3**	Concrete Risk Assessment	Offer visual aids and/or pamphlets of two varieties – one with only the most common adverse effects and one with all adverse effects – allow patient the option to view either based on their preference
**Theme 4**	Empathetic Individualization	Schedule longer visit time with patients you are meeting for the first time and allow time/space for patients to ask questions Inquire about patient’s social situation, work life, family, etc. Perform a thorough physical exam
**Theme 5**	Strong Preference for Oral Therapy	Explore with patients the option of upadacitinib, an oral alternative to infusions such as infliximab, as a rescue therapy while hospitalized and maintenance therapy as an outpatient

## Abbreviations

ASUC: acute severe ulcerative colitis
BID: twice daily
CDI: Clostridium difficile infection
CMV: cytomegalovirus
CRP: C-reactive peptide
CT: computer tomography
CYP: cytochrome P450
EIA: enzyme immunoassay
ESR: erythrocyte sedimentation rate
FDA: federal drug agency
Hgb: hemoglobin
IL: interleukin
IV: intravenous
JAK: Janus kinase
TID: three times daily
TNF: tumor necrosis factor
